# Repeated Long-Term DT Application in the DEREG Mouse Induces a Neutralizing Anti-DT Antibody Response

**DOI:** 10.1155/2016/1450398

**Published:** 2016-12-15

**Authors:** Junhua Wang, Myriam Siffert, Markus Spiliotis, Bruno Gottstein

**Affiliations:** ^1^Institute of Parasitology, University of Bern, Bern, Switzerland; ^2^Department of Infectious Diseases and Pathobiology, University of Bern, Bern, Switzerland

## Abstract

Regulatory T (Tregs) cells play an important role in mediating tolerance to self-antigens but can also mediate detrimental tolerance to tumours and pathogens in a Foxp3-dependent manner. Genetic tools exploiting the* foxp3* locus including bacterial artificial chromosome- (BAC-) transgenic DEpletion of REGulatory T cells (DEREG) mice have provided essential information on Treg biology and the potential therapeutic modulation of tolerance. In DEREG mice, Foxp3^+^ Tregs selectively express enhanced green fluorescent protein (eGFP) and diphtheria toxin (DT) receptor, allowing for the specific depletion of Tregs through DT administration. We here provide a detailed overview about an important consideration that long-term administration of DT induces a humoral immune response with an appropriate production of anti-DT antibodies that can inactivate DT and thus abrogate its effect in the DEREG mouse. Additionally, we showed that anti-DT mouse serum partially neutralized DT-induced Foxp3 inhibition.

## 1. Introduction

Regulatory T (Tregs) cells play an important role in mediating tolerance to self-antigens, and both their lineage and function are specifically defined by the transcription factor Foxp3 [1, 2]. Foxp3 specifies the Treg cell lineage and is crucial for immune tolerance against pathogens and tumour cells [3–6]. Foxp3 reporter mice have been essential in order to dissect the functions of Treg cells in vivo. One such mouse strain, DEpletion of REGulatory T cells (DEREG), makes use of a bacterial artificial chromosome (BAC) expressing a diphtheria toxin receptor (DTR) and enhanced green fluorescent protein (eGFP) fusion under control of the Foxp3 locus [7]. The vast majority of Foxp3^+^ Treg cells from DEREG mice are depleted in response to DT [7]. It was demonstrated that adult DEREG mice showed no observable signs of illness after Treg cell depletion, while newborn mice develop scurfy-like disease [7]. This has the unique advantage that the in vivo function of Treg cells can be studied in various settings in adult DEREG mice without the mortality associated with uncontrolled autoimmunity. So far, this model was mainly used to study a temporally transient dysfunction of Tregs. Thus, Treg inactivation strategies in view of studying a chronic and long-term DT administration have not been addressed yet, according to our literature search. Studies showed that following DT treatment of naïve DEREG mice, Treg depletion was transient with the frequency of Foxp3^+^ Tregs returning to wild type (WT) levels within 6 days. In addition, the newly emerged Foxp3^+^ Tregs no longer expressed the DTR-eGFP transgene, thus preventing prolonged depletion. Similar results were observed in mice undergoing either an acute or chronic viral infection. Furthermore, DT treatment in both transgenic DEREG mice and wild type (WT) mice resulted in enhanced morbidity and mortality [8]. In preliminary (unpublished) experiments using a murine infection model for alveolar echinococcosis (larval infection with the fox tapeworm* Echinococcus multilocularis*), we were facing unexpected findings concerning the time span of DT administration and the duration of the expected effect of Treg inactivation. In this context, we raised the question if long-term administration of DT could (i) induce a humoral immune response with an appropriate production of anti-DT antibodies that could inactivate DT and thus (ii) abrogate its effect in the DEREG mouse.

## 2. Material and Methods

### 2.1. Mice

Male DEREG mice, kindly provided by Professor Manfred Kopf (ETH, Zurich, Switzerland) with the agreement of Professor Tim Spawasser (TWINCORE, Hannover, Germany), were backcrossed to wild type C57BL6 at the animal facility of the Institute of Parasitology, University of Bern (Bern, Switzerland). All the offsprings were genotyped by PCR with specific primers for GFP and DTR. Foxp3^+^ Treg cell depletion was achieved by intraperitoneal (i.p.) administration of 110 ng diphtheria toxin (DT) (A) 3 times per week for 1, 2, 3, and 4 weeks. DT administration was stopped after 4 weeks; Foxp3 and anti-DT antibody were determined at indicated time-points. (B) Titrated DT was injected i.p. at 0, 75, 150, and 300 ng; Foxp3 was determined by flow cytometry at day 1 and day 3 after DT injection. (C) 110 ng DT was injected i.p. 3 times per week at 1, 2, 3, and 4 weeks and then stopped. Foxp3 was determined by flow cytometry at week 4 and week 16.

The animal study was performed in strict accordance with the recommendations of the Swiss Guidelines for the Care and Use of Laboratory Animals. The protocol was approved by the Commission for Animal Experimentation of the Canton of Bern (approval number BE_103/11).

### 2.2. Flow Cytometry

Aliquots of 10^5^ spleen cells/100 *μ*L of staining buffer per well were incubated each with 1 *μ*g of purified anti-CD16/CD32 for 20 min in the dark in order to block nonspecific binding of antibodies to the Fc*γ*III and Fc*γ*II receptors. Subsequently these cells were stained with surface marker separately for 15 min with 1 *μ*g of PE-Cy5-labelled anti-CD4 (GK1.5, eBioscience, San Diego, USA). For intracellular staining, spleen cells were first incubated with Inside Fix for 20 mins at room temperature and then stained with PE-labelled anti-Foxp3 (BD Pharmingen, Palo. Alto, CA, USA) in Inside Perm for 15 min in the dark. Corresponding PE-labelled rat IgG2a (kappa chain) was used as isotype control. Cells resuspended in 300 *μ*L of buffer (0.15 M NaCl, 1 mM NaH_2_PO_4_ H_2_O, 10 mM Na_2_HPO_4_ 2H_2_O, and 3 mM NaN_3_) were analysed in a flow cytometer (Becton Dickinson, Heidelberg, Germany) using the corresponding CELL QUEST software.

### 2.3. Enzyme-Linked Immunosorbent Assay (ELISA) for Anti-DT Antibody Detection

ELISA plates (Nunc Immulon) were coated with 100 *μ*L DT (Calbiochem, San Diego, CA, USA) solution (5 *μ*g DT/mL conventional carbonate/bicarbonate coating buffer) overnight at +4°C. After washing coated plates 3 times with Tris-Tween buffered saline (TTBS), serum samples (100 *μ*L, 1 : 50 dilution in TTBS plus 1 mg/mL bovine haemoglobin) were added to each well, and after 1-hour incubation at 37°C and three washes with TTBS, the wells were incubated with alkaline phosphatase-conjugated anti-mouse antibody (1 : 1000 dilution in TTBS, Lot number 0000103573, Promega, Madison, WI USA). Alkaline phosphatase substrate (1 mg NPP/mL, Lot number 1001595631, Sigma, Spruce St., USA) was then added, and absorbance was measured at 405 nm using a Tecan Sunrise® plate reader (Tecan, Grödig, Austria).

### 2.4. Anti-DT Serum Blocking

Six wild type C57BL6 mice were injected 110 ng DT i.p. 3 times per week and kept for another 4 weeks. Mice were sacrificed and blood was taken by heart puncture. Serum levels of anti-DT antibody were detected by ELISA, as described above. All serologically positive sera were pooled. From this serum-pool, 200 *μ*L was i.p. injected per mouse, and 110 ng DT was i.p. injected per mouse one day later. All animals were sacrificed at the 3rd day after DT injection, and CD4^+^Foxp3^+^ frequency was measured in spleen cell suspensions by using flow cytometry.

### 2.5. Statistical Analyses

All data were analyzed by SPSS 17.0. The results are presented as means ± SD. Normality of data was assessed by D'Agostino and Pearson and Shapiro-Wilk test. For normally distributed groups pf data, one-way ANOVA or unpaired two-tail Student's *t*-test was used to compare the differences between groups. Significance was defined as *P* < 0.05 for all tests.

## 3. Results

### 3.1. Long-Term DT Treatment

**Figure 1:**
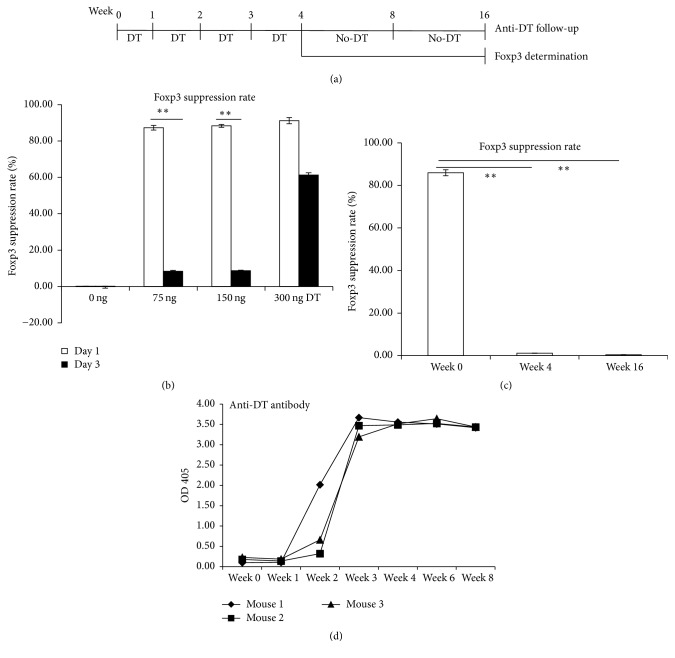
Experimental design, Foxp3, and anti-DT antibody follow-up during chronic DT treatment. (a) Schematic presentation of the experimental design. Foxp3^+^ Treg cell depletion was achieved by intraperitoneal (i.p.) administration of 110 ng diphtheria toxin (DT) 3 times per week for 1, 2, 3, and 4 weeks. DT administration was stopped after 4 weeks; Foxp3 and anti-DT antibody were determined at indicated time-points. (b) Titrated DT was injected i.p. at 0, 75, 150, and 300 ng; Foxp3 was determined by flow cytometry at day 1 and day 3 after DT injection. (c) 110 ng DT was injected i.p. 3 times per week at 1, 2, 3, and 4 weeks and then stopped. Foxp3 was determined by flow cytometry at week 4 and week 16. (d) Serum levels of anti-DT antibody were determined by using ELISA at indicated time-points. The mice were aged 8 weeks when used to start the initial DT treatment. ^*∗∗*^*P* < 0.01.

In a first experiment, “titrated” DT was intraperitoneally (i.p.) injected at 0, 75, 150, and 300 ng DT per mouse, and Foxp3 was measured at day 1 and day 3 after DT injection. Flow cytometric analyses showed that Foxp3 was largely depleted by DT at 75 and 150 ng at day 1 (Foxp3 suppression rate of 87.3% and 88.3%, resp., Figure 1(b)) but largely recovered at day 3 (Foxp3 suppression rate of 8.4% and 8.7%, resp., Figure 1(b)), while there was only a partial recovery at 300 ng DT at day 3 (Foxp3 suppression rate of 61.3%, Figure 1(b)). Mice were then treated with 110 ng DT/mouse and 3 times per week and subsequently sacrificed at 4 and 16 weeks after treatment, respectively. Flow cytometric analyses showed that Foxp3 was not depleted at week 4 and week 16 following DT administration (Figure 1(c)).

### 3.2. Anti-DT Antibodies Were Produced 2 Weeks after DT Treatment

To explore why Foxp3 was not depleted with a continuous DT administration, that is, 4 weeks after initiation of treatment, we examined serum levels of anti-DT antibodies at different time-points. ELISA results showed that, 2 weeks after DT treatment, serum levels of anti-DT antibodies became detectable in all of the three mice, with, however, a clear variation in the individual levels of anti-DT antibodies concentrations (OD_405_ 2.016 versus 0.110). Three weeks after DT administration, serum levels of anti-DT antibodies were significantly higher in all of three mice and maintained a high level even another 4 weeks after having stopped DT administration, when compared to the levels before DT administration (OD_405_  3.44 ± 0.24 at week 3 versus 0.17 ± 0.07 at week 0) (Figure 1(d)).

### 3.3. Anti-DT Serum Partially Neutralized DT-Induced Foxp3 Inhibition

**Figure 2:**
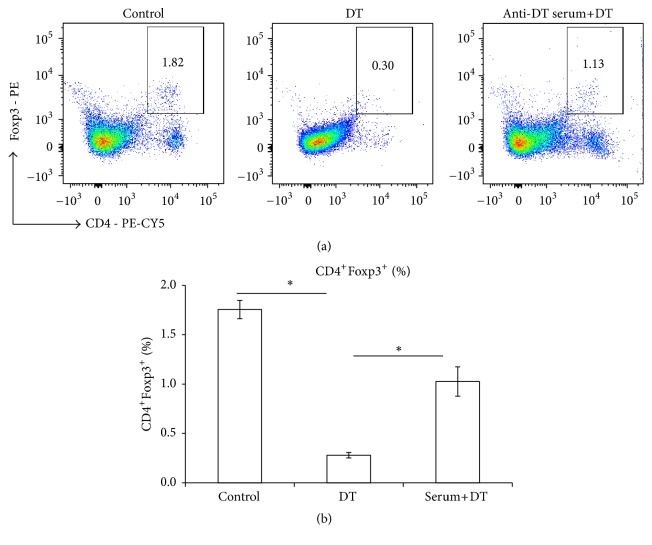
Anti-DT serum blocking. (a) Representative images of Foxp3^+^ T cells within CD4^+^ T cells with/without anti-DT serum blocking. (b) Frequency of Foxp3^+^ T cells within CD4^+^ T cells with/without anti-DT serum blocking. Six wild type C57BL6 mice were injected 110 ng DT i.p. 3 times per week and maintained for another 4 weeks. Mice were sacrificed and blood was taken from heart puncture. Serum anti-DT antibody levels were detected by ELISA. Seropositive samples were pooled, and from this pool 200 *μ*L anti-DT serum was injected i.p. per mouse, and subsequently 110 ng DT/mouse was injected i.p. one day later. All the animals were sacrificed at the 3rd day, and CD4^+^Foxp3^+^ frequency in the spleen was measured by flow cytometry. ^*∗*^*P* < 0.05.

To further study whether anti-DT antibodies could neutralize DT-induced Foxp3 inhibition, we produced an anti-DT serum (pool of three DT-treated animals), and aliquots of this serum-pool were injected to DEREG mice one day before DT application. Flow cytometry results showed that anti-DT antibodies could partially neutralize DT-induced Foxp3 inhibition, with Foxp3 percentage at 1.95 ± 0.11% (control), 0.26 ± 0.07% (with DT administration), and 1.02 ± 0.09% (DT administration + anti-DT serum injection), respectively (Figures 2(a) and 2(b)).

## 4. Discussion

In recent years, immunological studies within infectious disease models have increasingly focused on the pathogen-induced modulation of Treg function as a tool to improve the survival potential of the infectious organisms [9, 10]. For a long time specific in vivo targeting of Tregs was hampered by the lack of appropriate markers. The discovery of Foxp3 as a Treg-specific transcriptional factor enabled the development of Treg-specific mouse models. In DEREG mice (DEpletion of REGulatory T cells), a BAC (bacterial artificial chromosome) transgenic mouse line allows direct in vivo analysis and depletion of this exceedingly important cell type [8]. DEREG mice carry a DTR-eGFP transgene under the control of an additional Foxp3 promoter, thereby allowing specific depletion of Tregs by application of diphtheria toxin at any desired point of time during an on-going immune response. Adult DEREG mice, depleted of Treg cells, lack signs of autoimmune response [8]. Notably, this allowed for the broad use of DEREG mice to document the involvement of Treg cells in immune regulation and to gain insights into Treg cell biology. The protection from autoimmunity despite efficient transient Treg cell depletion is therefore not a limitation but an advantage of DEREG mice [8]. Anticipated health side effects of DT applied to DEREG mice as well as its efficiency to affect Treg function should be controlled by careful dose titration of the DT treatment in WT mice, previous to performing large-scale studies in knock-out animals. From previous studies we could learn that Treg cells were depleted by intraperitoneal injection of DT either at a high dose (1 *μ*g DT/mouse), with an application for three consecutive days [11], or medium dose (25 *μ*g/kg body weight, approximately 0.5 *μ*g DT/mouse) for one (days 0 and 1) or three rounds on two consecutive days (days 0, 1, 7, 8, 14, and 15) [12], or an initial dose of 200 ng DT and 100 ng DT every third day for 15 days [13]. However, a long-term DT administration that is persisting for 1 month or up to 4 months with a low dose of DT has never been studied, even though it may be very interesting to study the role of Treg in vivo concerning chronic disease models such as alveolar echinococcosis, where the parasite requires many months to fully develop and reach fertility. In our present study, we showed the possibility to treat DEREG mice with a low dose (110 ng DT/mouse, 3 times/week) for 1 to 4 months without affecting the health status of mice. However, while first conventionally depleted, Foxp3 became undepleted within 1 month of DT administration, accompanied in parallel with a high level of anti-DT antibody production by the treated animals. Subsequently we could evidence that anti-DT antibodies partially abrogate DT-induced Foxp3-inhibition by neutralizing DT in the mice, with the result that Foxp3 became only partially depleted after long-term DT administration. Teh and Gray discussed an interesting point that could explain the mechanism that ensures that the Treg niche is rapidly refilled following depletion [14]. These Treg cells could be (i) the product of peripheral conversion of activated conventional cells to peripherally induced Treg (pTreg) cells or (ii) the product of a homeostatic proliferation of existing thymus-derived Treg (tTreg) cells. Evidence for the former is supported by the authors' observation that there was a 20% reduction in Treg cells expressing neuropilin-1 (a cell surface marker used to distinguish tTreg (neuropilin-1 high) from pTreg (neuropilin-1 low) cells [15]), in the replenished Treg niche, as compared to the original Treg cell population [14]. However, it is noteworthy that neuropilin-1 may not be a reliable marker for pTreg cells under inflammatory conditions [15], which, however, seems to have been the case in their DT-treated mice [14]. On the other hand, a separate study found that homeostatic expansion of Treg cells (through proliferation and reduced apoptosis) could completely restore the compartment following 50% Treg cell depletion, with no evidence of conversion of conventional T cells observed [16]. This mechanism could be further confirmed due to neutralizing effects regarding DT administration and Foxp3 inhibition.

## 5. Conclusions

In conclusion, our findings indicate that Foxp3 cannot be kept knocked-down for a longer period of DT administration due to subsequent anti-DT antibody synthesis by treated animals and DT-neutralization via DT-specific antibodies. These observations suggest that Foxp3 levels and anti-DT antibody levels have to be carefully monitored in every situation where DT treatment is used for more than 2 weeks.
